# Imaging Appearances in Gout

**DOI:** 10.1155/2013/673401

**Published:** 2013-03-25

**Authors:** Gandikota Girish, David M. Melville, Gurjit S. Kaeley, Catherine J. Brandon, Janak R. Goyal, Jon A. Jacobson, David A. Jamadar

**Affiliations:** ^1^Department of Radiology, University of Michigan, 1500 East Medical Center Drive, TC 2910, Ann Arbor, MI 48109-0326, USA; ^2^Division of Rheumatology, University of Florida College of Medicine, Jacksonville, FL 32209-6561, USA; ^3^Division of Rheumatology, Raritan Bay Medical Center Perth Amboy, NJ 08861, USA

## Abstract

Gout is an ancient disease. Last decade has brought about significant advancement in imaging technology and real scientific growth in the understanding of the pathophysiology of gout, leading to the availability of multiple effective noninvasive diagnostic imaging options for gout and treatment options fighting inflammation and controlling urate levels. Despite this, gout is still being sub-optimally treated, often by nonspecialists. Increased awareness of optimal treatment options and an increasing role of ultrasound and dual energy computed tomography (DECT) in the diagnosis and management of gout are expected to transform the management of gout and limit its morbidity. DECT gives an accurate assessment of the distribution of the deposited monosodium urate (MSU) crystals in gout and quantifies them. The presence of a combination of the ultrasound findings of an effusion, tophus, erosion and the double contour sign in conjunction with clinical presentation may be able to obviate the need for intervention and joint aspiration in a certain case population for the diagnosis of gout. The purpose of this paper is to review imaging appearances of gout and its clinical applications.

## 1. Introduction

Gout is the most common cause of inflammatory arthritis in men [1] and its prevalence is rapidly expanding in the general population [2]. It is associated with an excess of uric acid in the body. This results in supersaturation of uric acid in body tissues and fluids resulting in urate deposition. Over 80% of the gout patients have a positive family history of gout or hyperuricemia. The disease is best understood as having four phases which include asymptomatic hyperuricemia, acute, intercritical, and chronic gout. The musculoskeletal manifestations of gout are triggered by the deposition of monosodium urate (MSU) crystals in cartilage, joints, and soft tissues. Acute gout attacks are due to the triggering of an inflammation pathway known as the NALP3 inflammasome by MSU crystals in the joint [3] and soft tissues. The diagnosis of gout is confirmed by the presence of intracellular MSU crystals in a joint aspirate [4]. MSU crystals are not radioopaque and are identified on polarized microscopy as negatively birefringent. Chronic gout can take years to develop and its findings include chronic synovitis, tophus formation, and erosions. Thus, the crystal induced tissue reaction in gout is different from other types of inflammatory arthritis where synovial inflammation is thought to be the predominant primary cause of tissue damage.

An experienced clinician or a specialist in gout can make the diagnosis on clinical grounds and laboratory findings and provide optimal management with little or no help from imaging, except in certain cases where the presentation mimics mass lesions or infection or when the deeper structures like the spine and sacroiliac joints are involved. However, a majority of patients with gout present to and are being cared for by nonspecialists, and the management remains suboptimal [5–7]. In such scenarios, imaging may have a helpful adjunct role in the diagnosis and management of gout, for the inexperienced provider. The awareness of recent advances in the imaging of gout, specifically in the field of high-frequency, high-resolution ultrasound (US) and dual energy computer tomography (DECT) will help clinicians use imaging where appropriate and for the sonographers and radiologists to be more confident in the diagnosis of gout. The aim of this paper is to review and familiarize the reader with the imaging (radiographs, US, computed tomography (CT), DECT, and magnetic resonance imaging (MRI)) and findings of gout.

## 2. Imaging

Common imaging findings of gout are described in Table 1. Comparative utility of X rays, US, CT and MRI in the diagnosis of gout is discussed in Table 2. Advanced imaging is very sensitive in demonstrating aggregates of MSU crystals in soft tissue, joint, and bone. The extent and distribution of the crystal deposits have been greater than previously thought. The previous misconception is likely due to the fact that MSU crystals dissolve in formalin, and therefore, were not routinely identified in fixed pathological specimens. In addition, areas of crystal deposition were not routinely examined during autopsies.

The surface of joint cartilage and most tendons and ligaments are well shown by sonography. MSU crystals in tophaceous deposits around joints and deposits in tendons and soft tissues are well identified by DECT. CT can clearly demonstrate tophi growing into the adjacent bone, causing joint erosions with over hanging margins. MRI is the only clinical imaging modality which accurately shows bone marrow edema. Both ultrasound with Doppler imaging and MRI with contrast show increased vascularity associated with inflammation surrounding crystal deposits, sometimes even during intercritical periods.

Imaging is diagnostic in identifying tophi presenting as mass lesions or with symptoms of significant limitation of movement and pain, in superficial soft tissues (like patellar tendon, ankle tendons, and carpal tunnel) and deeper (like cruciate ligament in the knee and spine) structures. Tophi involving the flexor tendons at the carpal tunnel are well detected by ultrasound [8]. These tophi resolve with proper serum uric acid lowering treatment [9], and the progressive resolution can be followed by imaging.

## 3. Radiographs

**Figure 1:**
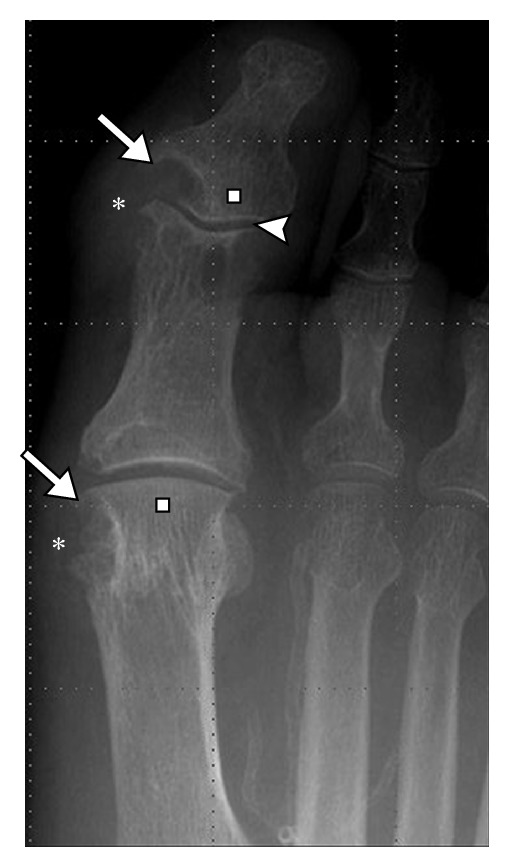
Gout on radiograph. Anteroposterior (AP) view of the 1st metatarsophalangeal (MTP) joint and interphalangeal joint demonstrating juxta-articular erosion with overhanging edge (long arrows). Note the relative preservation of joint space (arrowhead) and subchondral bone density (white square) involving the 1st MTP and interphalangeal joint. *Soft tissue tophus.

**Figure 2:**
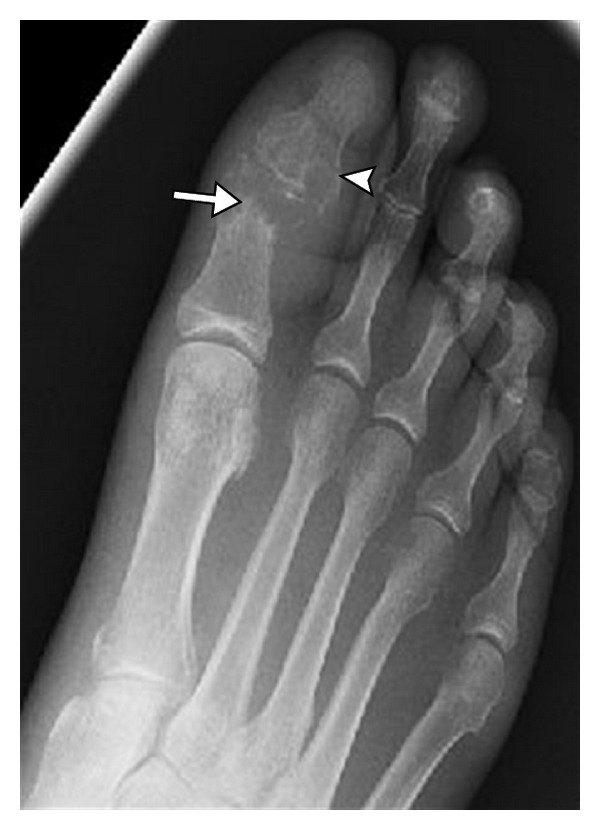
Subchondral gout. Anteroposterior view of the interphalangeal joint of the big toe showing subchondral deposition (long arrow) and associated erosive changes (arrowhead).

**Figure 3:**
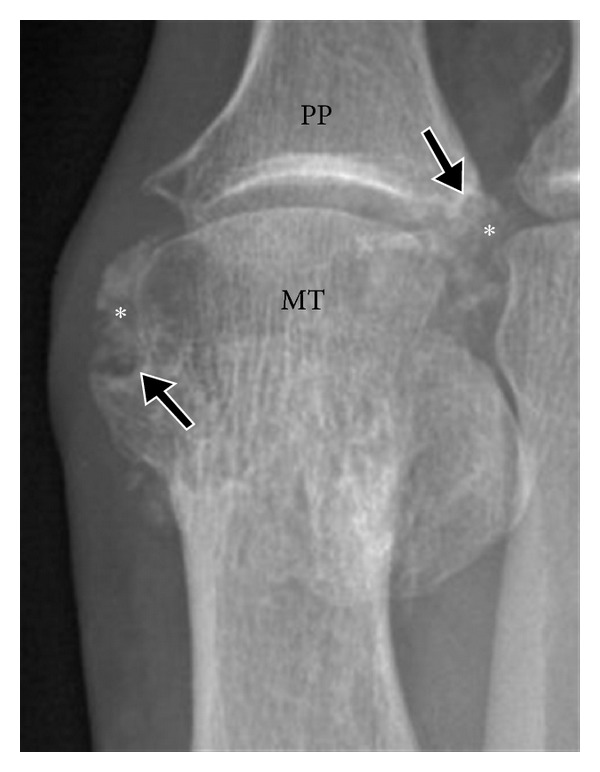
Tophaceous gout involving the 1st MTP joint. Anteroposterior radiograph shows a calcified soft tissue tophus (asterisk) with adjacent erosions (arrow). MT: first metatarsal head; PP: proximal phalanx. Possible associated calcium pyrophosphate or hydroxyapatite deposition must be considered.

Radiographic findings of gout occur late in the disease and underestimate the degree of involvement; hence, their role in diagnosis and management is limited. Characteristic radiographic findings of gout include, first MTP involvement (Figure 1), juxta-articular erosions with sclerotic margins and overhanging edges, and preservation of joint spaces and periarticular bone density until late in the disease process. McQueen et al. [10] proposed a cellular mechanism to explain the characteristic erosion appearances of overhanging edge. Osteoclasts are activated at the bone tophus interface, whereas osteoblasts are inhibited resulting in marked localized bone loss [10]. Gout deposits around joints can be juxta-articular, intra-articular, and subchondral (Figures 1 and 2) and usually do not demonstrate symmetric joint involvement. The tophus, the hallmark of chronic gout, is a soft tissue nodule representing the body's granulomatous immune reaction to MSU crystals [11] (Figure 2). Dense calcification in the tophus is a late finding and may be associated with disturbance in calcium metabolism (Figure 3). Erosions are often located next to a tophus (Figure 3).

## 4. Ultrasound

**Figure 4:**
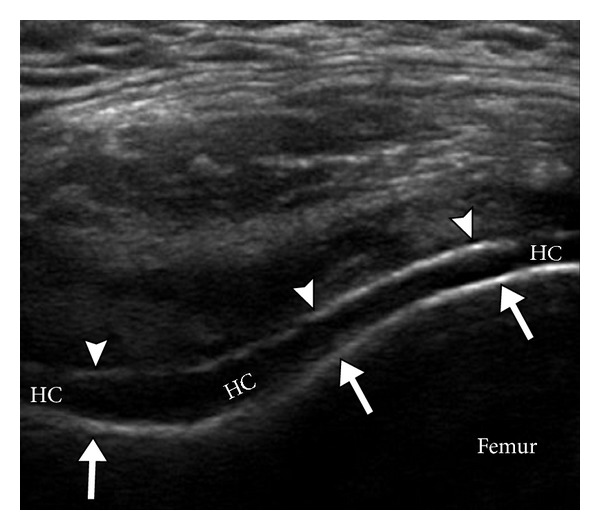
Ultrasound double contour sign. Transverse ultrasound image of the suprapatellar knee joint demonstrates two parallel hyperechoic contours on either side of the hypoechoic hyaline cartilage (HC). The deep echogenic contour (long arrows) represents the femoral cortex, while the superficial echogenic contour (arrowheads) represents uric acid crystals accumulating on the surface of the hypoechoic hyaline cartilage (HC).

Sonography is able to depict tophaceous deposits in soft tissues, joints, cartilage, as well as erosions, synovitis, and increased vascularity, without the use of contrast agents. Recent studies published support a positive role for US in the early diagnosis of gout and in monitoring treatment response [12, 13]. US may depict urate deposition over the most superficial layer of hyaline cartilage as an irregular echogenic line producing the “double contour sign” [14] (Figure 4). This sign has been noted in patients with an acute gout flare up, with a history of prior gout attacks, and with asymptomatic hyperuricaemia. The sensitivity of this finding ranges from 25% to 95% in patients with gout [15–19]. However, these studies are small, with varied study designs. It has been suggested that this sign can be seen as an early ultrasound finding in gout, even before the development of erosive changes. Further studies are needed to document the sensitivity and specificity of this sign in the early diagnosis of gout and its prognostic significance in patients with asymptomatic hyperuricaemia. 

**Figure 5:**
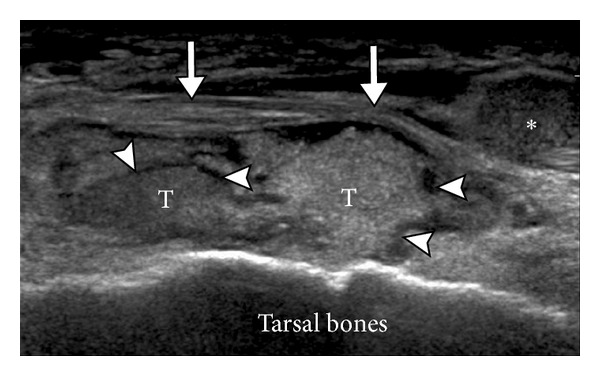
Tophus in gout. Ultrasound appearance of a tophus (T) overlying the dorsal aspect of the tarsal bones and underlying the extensor digitorum tendons (long arrows). Note the anechoic peripheral halo (arrowheads) and hyperechoic heterogeneous center. *Echogenic fluid.

The characteristic US appearance of a tophus includes an anechoic halo and hyperechoic heterogeneous center [16] (Figure 5). The peripheral anechoic halo likely represents the fibrovascular zone [20] noted in histology, with a more central hyperechoic synovial proliferation. Sometimes the tophus can be ill defined, traversing multiple fascial planes. Tophi that are sonolucent have been termed as “soft tophi” whilst long standing tophi that do not allow imaging of structures below them are termed as “hard tophi” [21].

**Figure 6:**
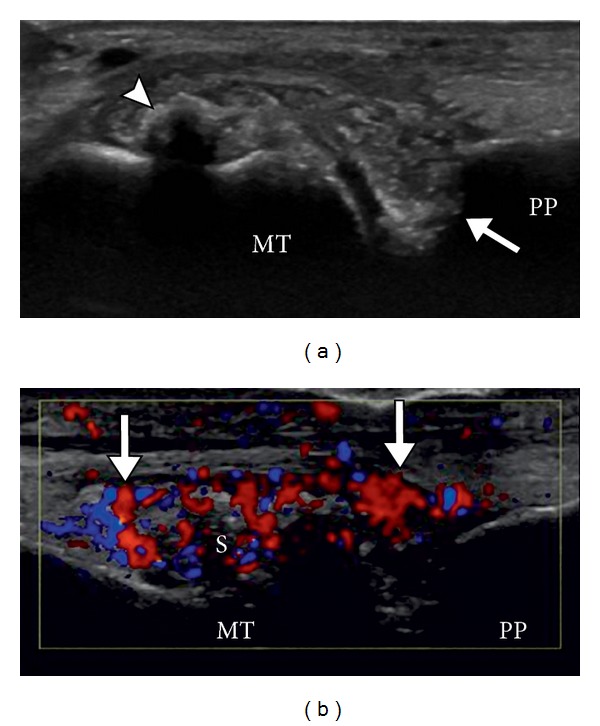
Gout with synovitis. Longitudinal US images of the 1st MTP joint without (a) and with (b) color Doppler show calcified, shadowing tophus (arrowhead) and adjacent heterogeneous soft tissue with associated hyperemia on color doppler imaging, consistent with synovial proliferation. Note the erosions at base of proximal phalanx (arrow).

**Figure 7:**
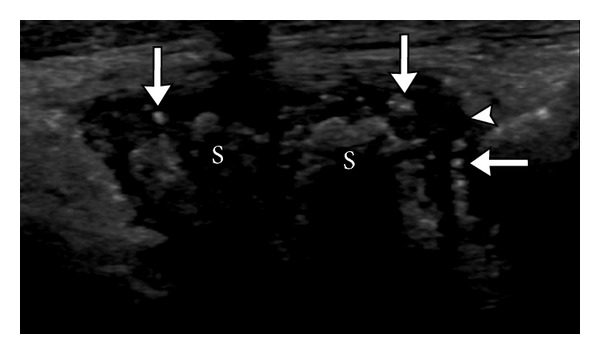
Snow storm appearance. Multiple hyperechoic foci (arrows) are noted in this first MTP joint floating in the anechoic joint effusion (arrowhead). Note the shadowing within the synovial thickening (S) within the joint, likely related to calcification. S: synovitis.

**Figure 8:**
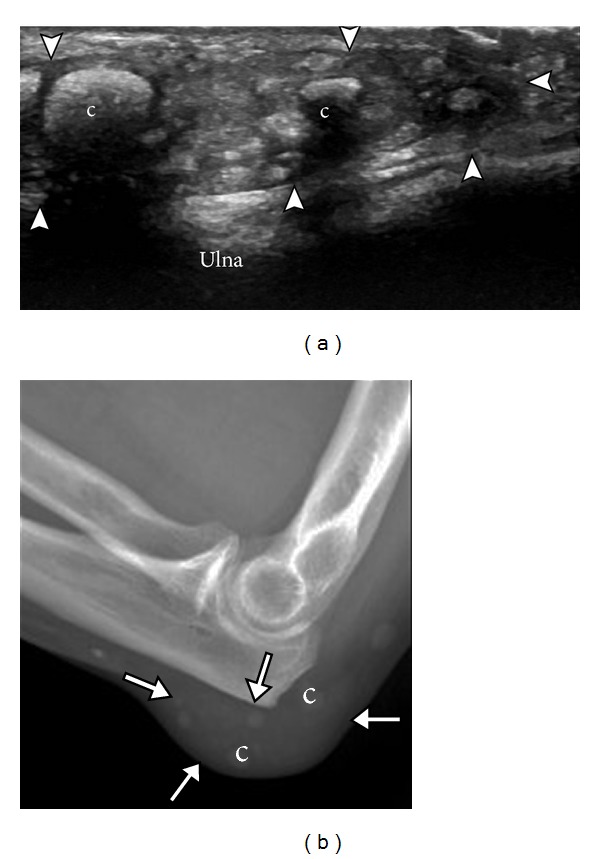
Olecranon bursa. Ultrasound (a) and radiograph (b) demonstrate olecranon bursa (arrowheads, arrows) overlying ulna. Note multiple soft tissue nodules in the bursa, some partially calcified (c).

**Figure 9:**
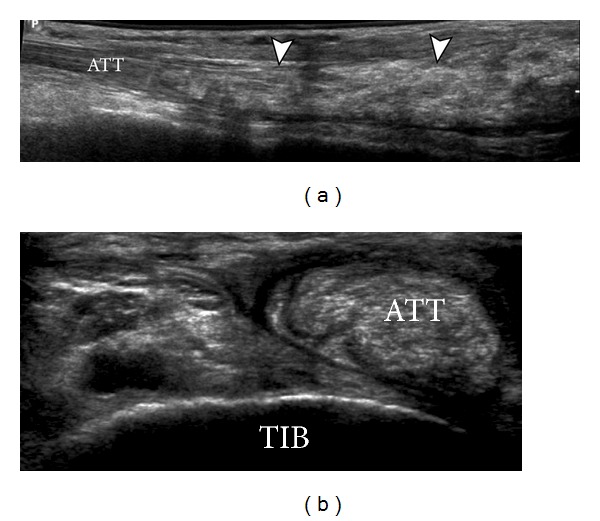
Gout depositions in tendons. Anterior tibialis tendon (ATT). Long (a) and short (b) axes views of ATT demonstrating hyperechoic gout deposit (arrowheads) within the substance of the distal ATT. TIB: tibia.

**Figure 10:**
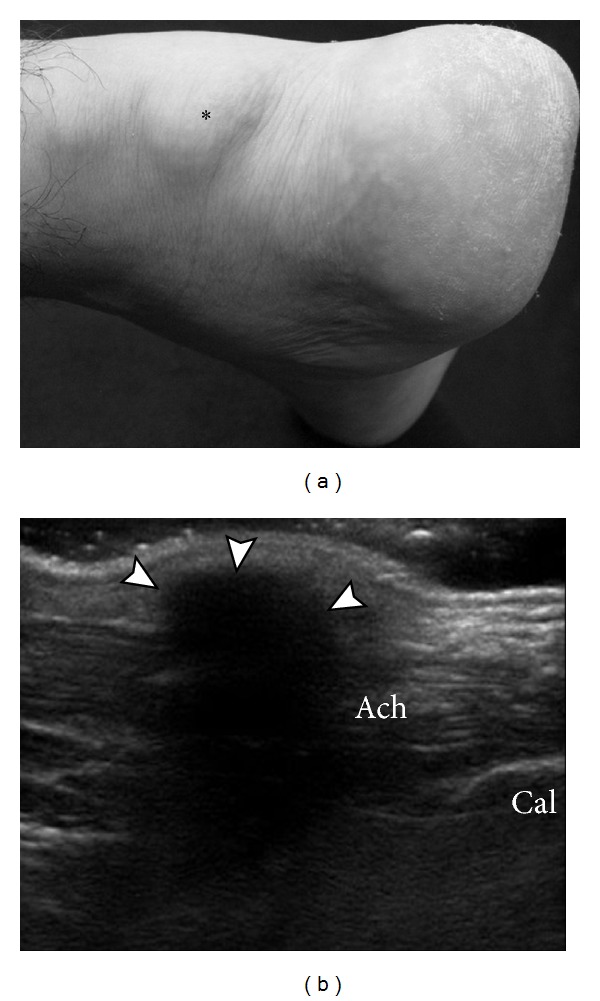
Subcutaneous tophaceous gout on US. Photograph (a) demonstrates soft tissue prominence (asterisk) without marked cutaneous inflammatory changes overlying expected location of the Achilles tendon. Longitudinal ultrasound image (b) shows densely shadowing echogenic focus overlying the Achilles tendon, consistent with subcutaneous tophus with peripheral calcification (arrowhead). Ach: Achilles tendon; Cal: calcaneus.

Synovitis in gout demonstrates mixed echogenicity on ultrasound, being predominantly hyperechoic and often associated with increased vascularity (Figure 6). It tends to be more concentric, unlike the frond-like synovial hypertrophy noted in rheumatoid arthritis [20]. In some cases, floating hyperechoic foci have been described, likely representing microtophi, resulting in “snow storm appearance” [22] (Figure 7). Ultrasound is excellent for identifying bursitis (Figure 8), intratendinous deposition (Figure 9), enthesitis, and subcutaneous nodules seen with gout (Figure 10). 

**Figure 11:**
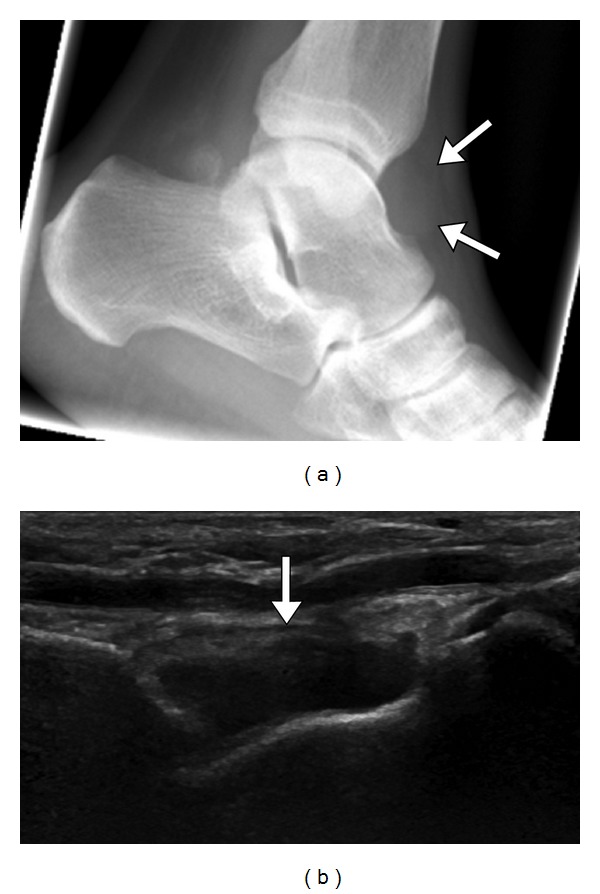
Tibiotalar gout with ankle effusion. Lateral ankle radiograph (a) shows ankle joint effusion (arrow). Longitudinal US (b) demonstrates moderate ankle joint effusion (arrow). Tibiotalar joint aspiration revealed crystals, confirming gout.

A joint effusion is an early but nonspecific finding in gout patients (Figure 11). Ultrasound is also the primary imaging modality used for needle guidance during diagnostic and therapeutic interventions, including aspirating fluid for crystals. Ultrasound may assist evaluation in acute gout, in not only identifying the extra-articular structure involved, but also allowing needle guidance for fluid aspiration. 

**Figure 12:**
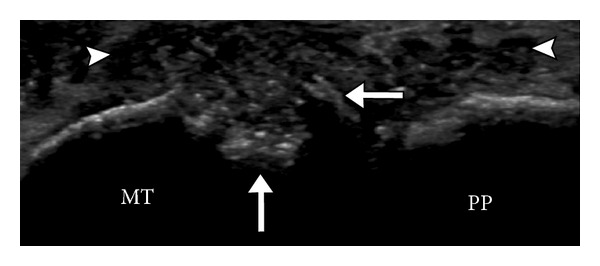
Synovitis and erosion. Ultrasound appearance of erosion in the metatarsal head demonstrates cortical irregularity, focal defect, and overhanging edge (arrows) with adjacent synovitis (arrowheads). MT: metatarsal; PP: proximal phalanx.

One pitfall of ultrasound imaging is its inability to image intraosseous gout. Caution must be used when diagnosing erosions with US. While it is true that US is more sensitive than radiographs for diagnosing erosions [19], US also can underestimate the extent and number of erosions, when compared to MRI [23]. The specificity of an ultrasound diagnosis of erosions is increased when there is adjacent synovitis or tophi (Figure 12) [24].

## 5. CT

**Figure 13:**
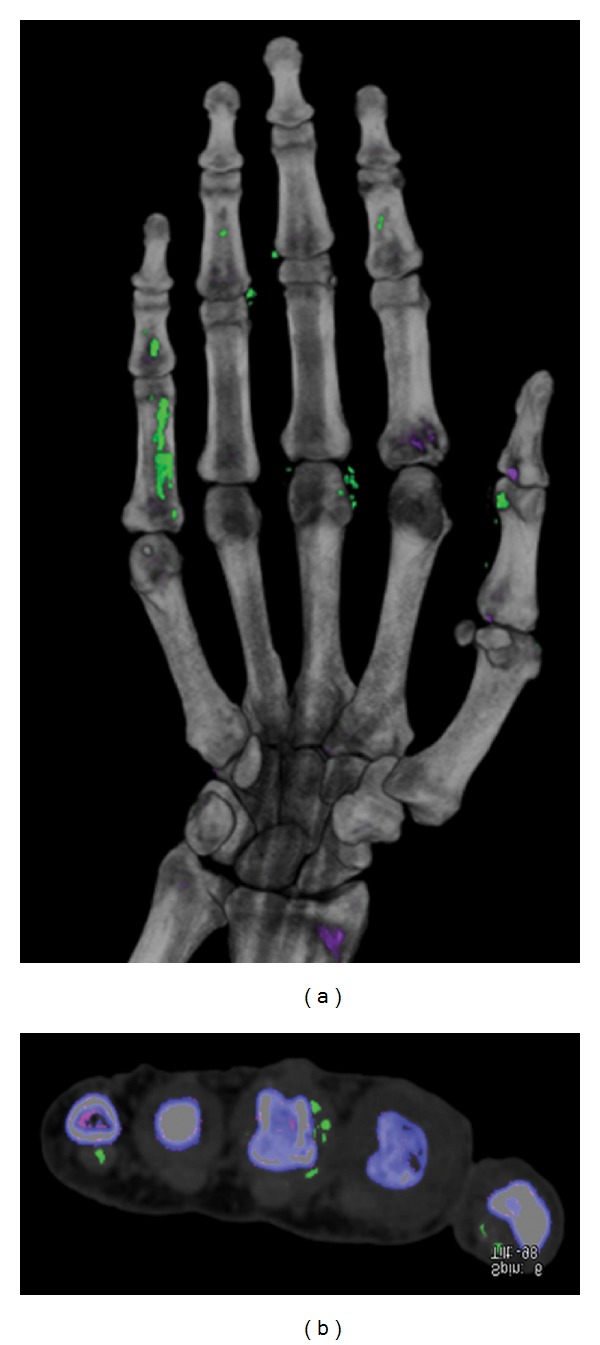
Dual energy computed tomography (DECT) images (a, b) of a hand showing tendinous and periarticular MSU deposition (color coded—green). (Courtesy Dr. K. Glazebrook, Mayo clinic, Rochester, MN, USA).

Dual energy computed Tomography (DECT) has an established role in the assessment of coronary artery plaques and uric acid calculi [25]. Its role in the diagnosis of gout is promising and evolving. Dual energy X-ray tubes at 80 kv and 140 kv are placed at 90 degrees to each other and to their two detectors. Images are acquired simultaneously. Based on the spectral dual energy properties, aggregates of urate crystals can be uniquely color coded, allowing for depiction and distinguishing alternative diagnosis, including other crystal deposition diseases, such as hydroxyapatite (Figure 13). This technique has a high accuracy in identifying cases of tophaceous gout and is very sensitive in detecting the volume of urate crystals relative to clinical examination [26]. Further studies are required to assess DECT's sensitivity and specificity in identifying very early nontophaceous gout without crystal aggregates (crystals less than 3 mm in size, microtophi, and crystals deposits on cartilage, etc.). DECT may be useful in evaluating patients with high clinical suspicion of tophaceous gout, in whom conventional diagnostic tests have been inconclusive. It may also help assess the presence of gout in atypical locations such as the spine. 

**Figure 14:**
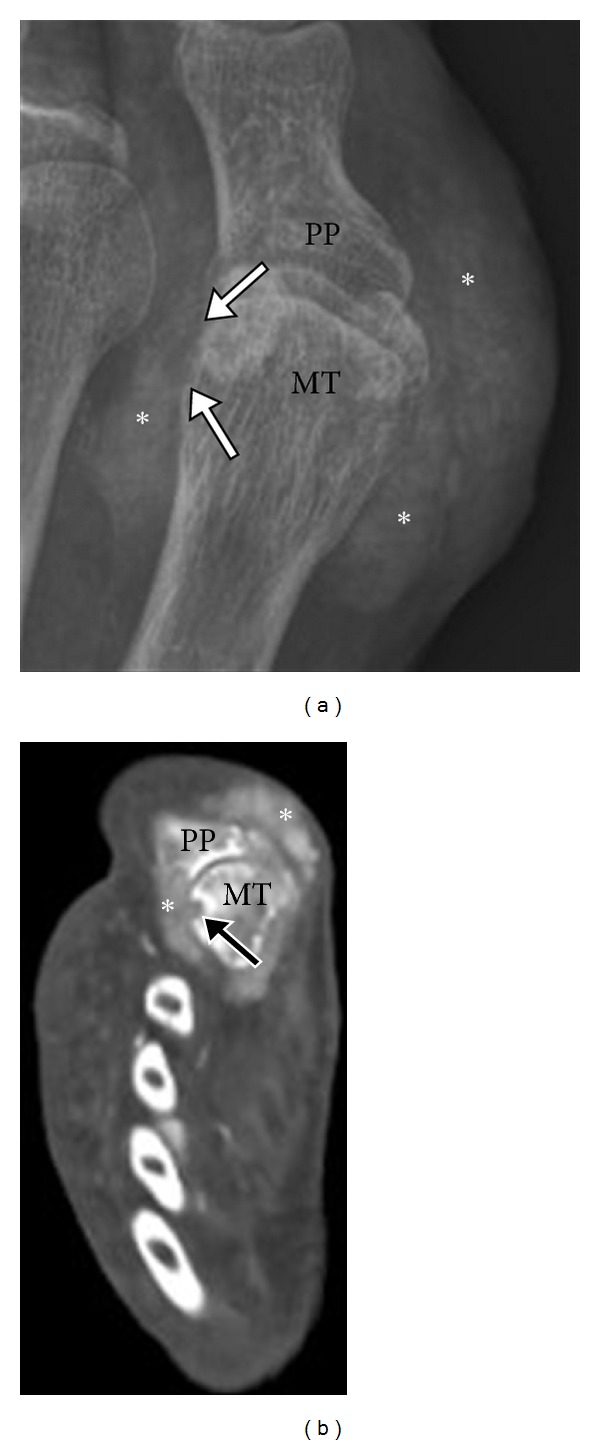
Tophaceous gout on CT. Anteroposterior radiograph (a) of the 1st MTP joint demonstrates dense soft tissue masses (*) centered on the 1st MTP with erosive changes involving the lateral aspect of the 1st MT head (arrows). Corresponding axial CT image (b) shows periarticular high attenuation soft tissue deposit adjacent to the first MTP joint (*) with focal cortical erosion (arrow). MT: metatarsal head; PP: proximal phalanx.

**Figure 15:**
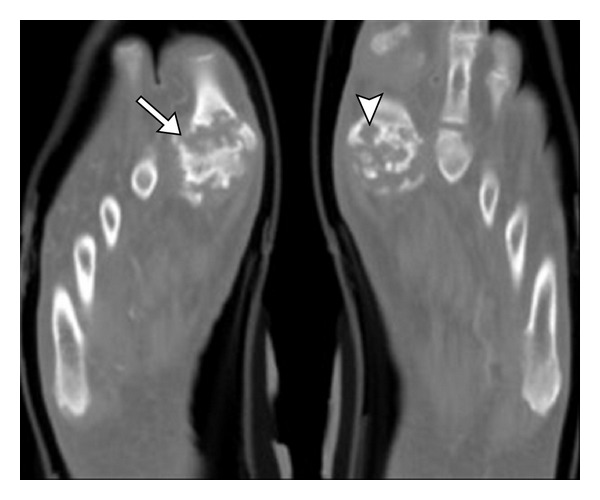
Advanced erosive gout on CT. Axial CT images of bilateral 1st MTP joints demonstrate severe erosive changes (arrow) related to chronic gout with intra-articular erosions and subchondral deposits (arrowhead). Note the preservation of bone density adjacent to erosions, a feature of gout.

Conventional CT is extremely sensitive in identifying characteristic gout erosions and tophus (Figure 14). Cost and radiation limit the routine use of CT. A tophaceous soft tissue nodule demonstrates a density of 170 Hounsfield units [27]. A tophus can be intra- (Figure 15) or extra-articular, as well as located in tendons and subcutaneous tissues, showing preponderance to the pressure points. Tophi are known to diminish in size in response to treatment, which can be documented by serial cross-sectional imaging. Even though CT and MRI are more accurate, US is probably more practical for follow-up studies because it is easily available, relatively of low cost, and has no ionizing radiation. 

## 6. MRI

**Figure 16:**
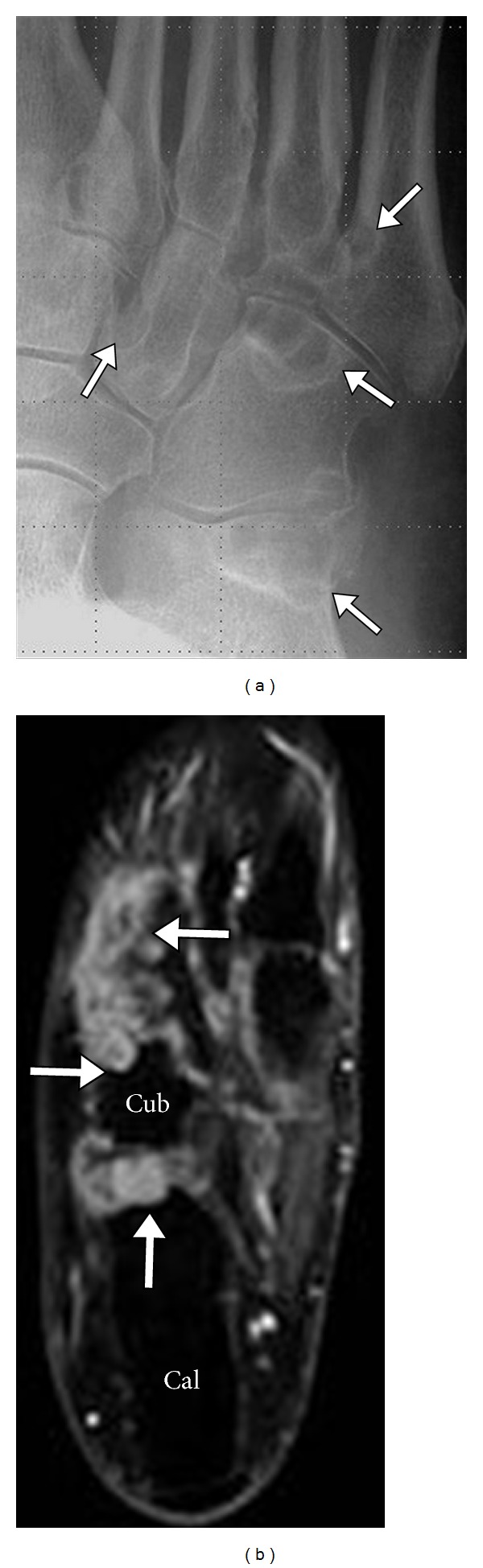
Intraosseous gout midfoot. Multifocal deposition of gout crystals in the tarsal bones of the mid foot (arrows) as seen in radiographs (a) axial (b) STIR MRI. Note the intermediate-to-high signal on STIR images and sclerotic margins on radiograph. Cal: calcaneus; Cub: cuboid.

**Figure 17:**
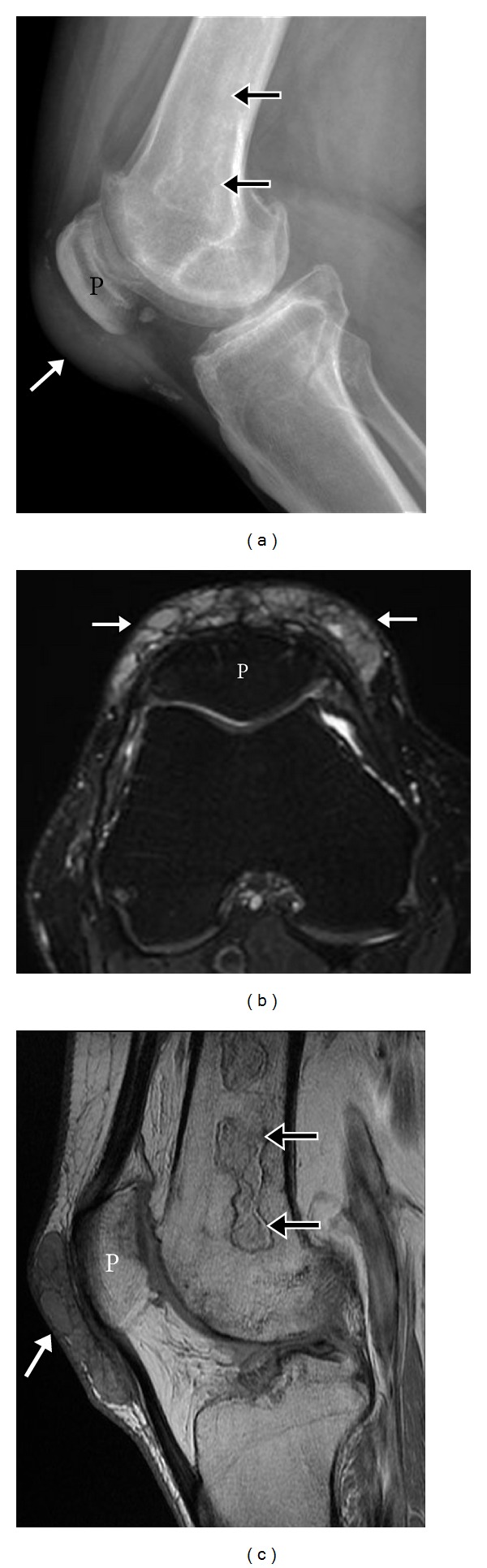
Prepatellar bursal gout. Lateral knee radiograph (a) shows dense focal prepatellar soft tissue swelling with peripheral soft tissue calcifications. Axial T2 (b) and sagittal fat saturated proton density (c) MR images demonstrate intermediate soft tissue corresponding to the prepatellar bursa with internal septations. Incidental note is made of femoral bone infarcts (thin arrow). P: patella.

**Figure 18:**
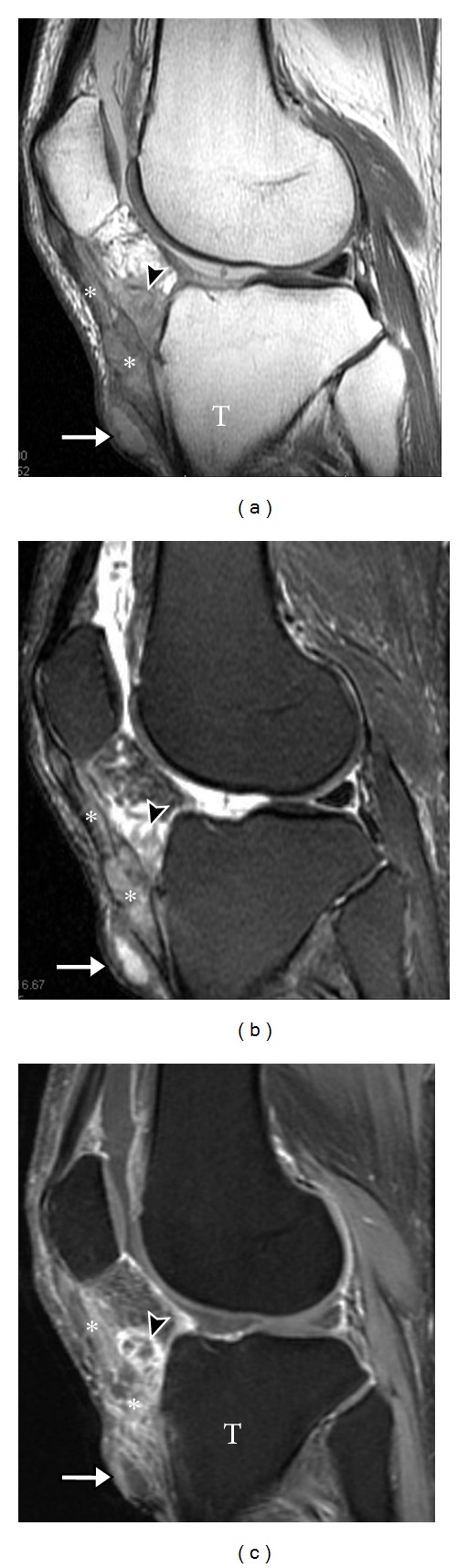
Intratendinous tophaceous gout on MRI. Sagittal T1 (a), sagittal T2 (b), and T1 postcontrast (c) MR images of the knee show abnormal, enhancing soft tissue gout deposit infiltrating the distal patellar tendon (asterisk) and extending across the facial planes to involve the adjacent Hoffa's fat pad (arrow head) and pretibial subcutaneous tissue (long arrow). T: tibia.

**Figure 19:**
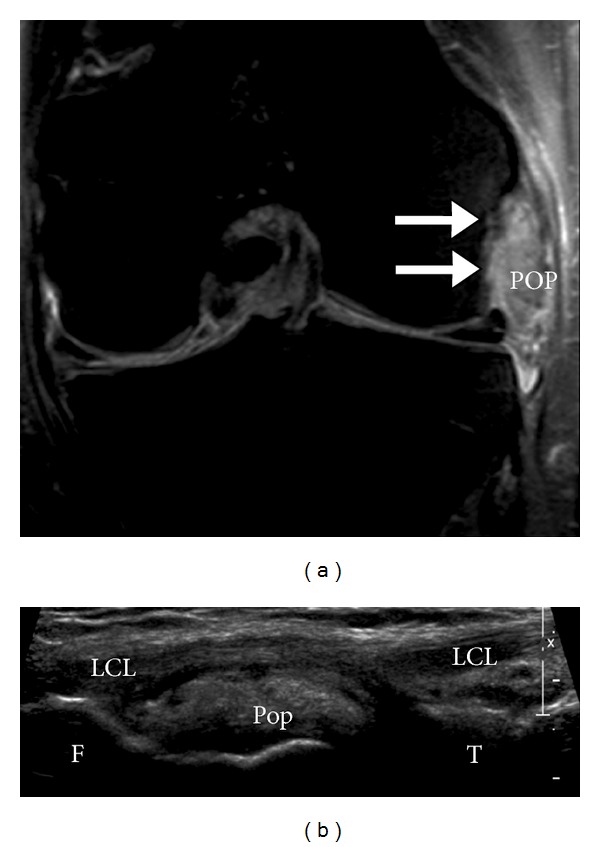
Gout involving intra-articular popliteus tendon in the knee. Coronal fat saturated T2 MR image (a) demonstrates intermediate signal gout deposit in the popliteus tendon (Pop) adjacent to the popliteus groove (arrows), deep to the lateral collateral ligament (LCL). Note the hyperechogenicity of the gout deposit on ultrasound (b). F: femur; T: tibia.

**Figure 20:**
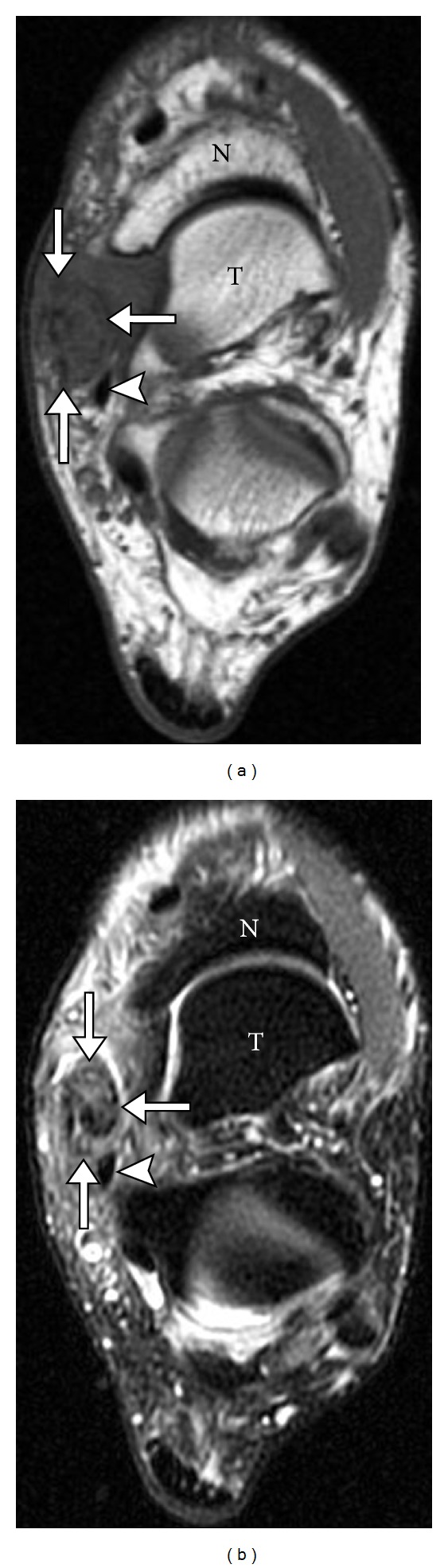
Gout involving tibialis posterior tendon. Axial T1 (a) and axial T2 (b) MR images show abnormal soft tissue infiltrating and surrounding the tibialis posterior tendon (arrows) adjacent to flexor digitorum tendon (arrowhead). Patient subsequently underwent surgery revealing complete rupture secondary to gout.

**Figure 21:**
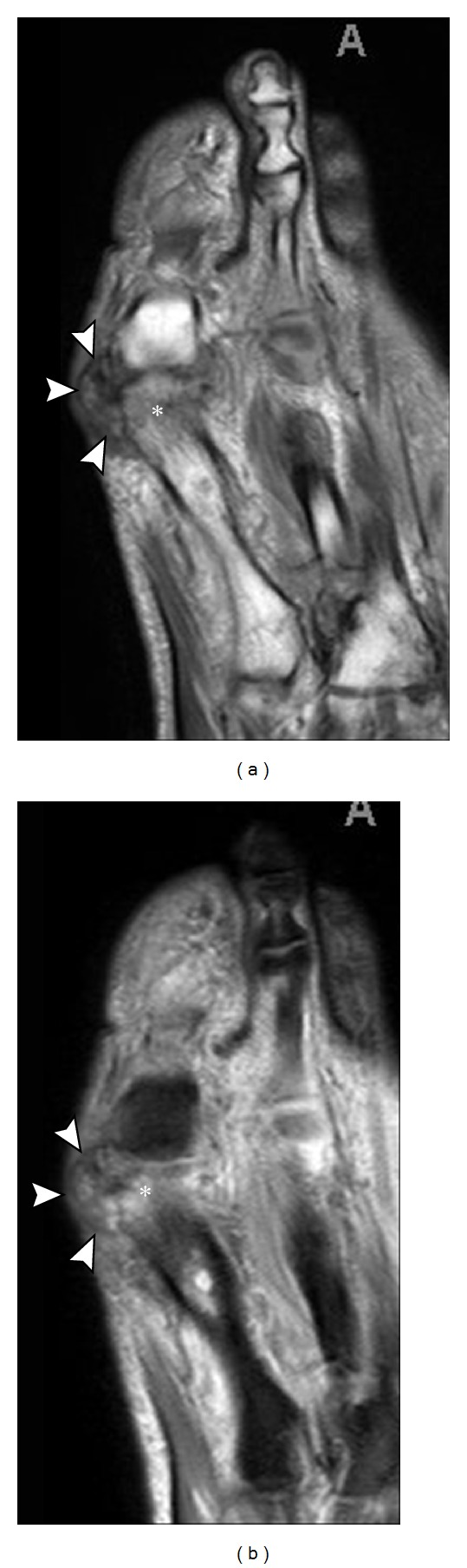
MRI appearances of tophus. Axial T1 (a) and T2 (b) MRI. Note the intermediate signal juxta-articular soft tissue mass (arrowhead), medial to the first MTP joint with marrow edema (asterisk) in the adjacent first metatarsal head.

MRI is helpful in the localization of gout deposit and can show gout in the deeper tissues like the spine and in locations not amenable to clinical examination, such as interosseous deposits in the midfoot (Figure 16). MRI is accurate in diagnosing the extent of gout involvement of the bursae and tendons, as well as any associated tendon tears (Figures 17, 18, 19, and 20). Tendon involvement by gout can mimic a mass lesion. A tophus histologically consists of central acellular crystalline core surrounded by “corona zone” and a peripheral “fibrovascular zone” [28]. Tophi on MRI are low signal on T1-weighted MRI and mostly intermediate signal on T2-weighted MRI (Figure 21). Some can be high signal on T2-weighted MRI and can show significant enhancement in postcontrast images. This enhancement would be proportional to the vascularity predominantly in the outer “fibrovascular zone” seen on histology [20]. Low signal foci on T2-weighted images most likely represent calcifications. 

## 7. Imaging to Differentiate between Gout, Other Crystalline Arthropathy, and Infection

Clinically, the presentation of gout can mimic an infection. Superimposed infection should always be considered. The characteristic appearance and location of the osseous erosion with gout and the absence of an adjacent soft tissue ulcer are helpful findings that suggest gout. However, joint aspiration with examination of fluid under plane polarized microscopy and gram stain and cultures are advisable.

MSU crystals deposit on the surface of the articular cartilage as an echogenic curvilinear band paralleling the cortex, giving the appearance of a “double contour sign” on ultrasound (Figure 4). This is a distinctly different pattern as compared to calcium pyrophosphate crystal disease which usually results in crystal deposition within the cartilage rather than the surface [14]. US is the most sensitive modality to pick up these differences.

## 8. Imaging in the Monitoring of Response to Treatment

With the advent of new and very effective treatment options for lowering urate levels in gout [29], there is a growing research interest in imaging to monitor treatment response. Such imaging changes include diminishing tophus size, disappearance of the “double contour sign,” and resolution of synovial hypertrophy, joint effusion, and bone marrow edema. Advanced 3D rendering of the tophus is now possible with both CT and MRI with CT considered more accurate and reproducible. DECT will identify the urate crystals, based on chemical composition, and will be more definitive and reliable in the followup of resolving tophus. MRI will retain its edge in following resolving synovial proliferation and bone marrow edema; however, ultrasound is an excellent and affordable alternative assessment method for all of the above imaging findings except marrow edema and provides fine details with excellent spatial resolution. While MRI is equally helpful in monitoring disease progression, for both clinical and research purposes, it is less readily available and more expensive. Therefore, ultrasound promises to be the modality of choice to monitor treatment response. 

## 9. Conclusion

The role of imaging in the management of long standing gout is usually limited, except when looking for gout deposits in the deeper tissues, where sampling can be challenging. Sonography can be used for needle guidance to obtain tissue samples for diagnosis. Recent advances in the imaging of gout show promise and hopefully will lead to more accurate assessment of the activity of gout and assist in the diagnosis of atypical presentations of acute and tophaceous gout, including its response to therapy. The significance of asymptomatic hyperuricaemia with positive early imaging findings is still to be determined. Since ultrasound is a readily available, nonionizing modality which can depict many features of gout, as well as assist with needle guidance, it may be a preferred modality for imaging gout. 

## Figures and Tables

**Table 1:** Common pathological findings of gout.

Erosions	Juxta-articular cortical irregularity and depression +/− overhanging edge +/− sclerotic margins
	Findings seen in at least two planes
	Erosions adjacent to tophus (causative agent) CT is most sensitive. US can overestimate

Synovial proliferation	Synovial thickening +/− enhancement on post contrast images +/− increased vascularity on Doppler imaging
	Both US and MRI are very sensitive
	Vascularity may not be obvious when patient is on treatment/NSAID Synovial proliferation gout ≪RA, needs more research

Tophus	Eccentric high-density soft tissue swelling from chronic granulomatous response to MSU crystals
	Can be intra- or extra-articular
	Characteristic US appearance: hypoechoic peripheral rim/halo and hyperechoic/heterogeneous center Can also be imaged by radiograph, DECT, CT, and MRI Calcification in the tophus suggests renal impairment

Bone marrow edema	Uncommon/minimal, specifically centered around erosion
	If extensive, think of inflammatory arthritis or infection, whether associated with the underlying diagnosis or not
	Only MRI can demonstrate bone marrow edema

Cartilage involvement	MSU crystals deposit on articular cartilage surface (anechoic curvilinear band paralleling the cortex) giving “double contour sign”
	Hydroxyapatite deposition is within cartilage substance US is most sensitive

Joint effusion	Anechoic fluid in the joint recess/space not specific sign unless accompanied by small numerous hyperechoic foci +/− “snow storm appearance”
	Aspirate to confirm gout and exclude infection

**Table 2:** Comparative utility of X-ray, US, CT, and MRI in the diagnosis of gout.

	X-ray	US	CT	MRI
Erosion	+	++	+++	++
Effusion	+	+++	++	+++
Synovial proliferation	−	+++	+	+++
Tophus	+	+++	++	+++
Joint space narrowing	+++	−	+++	+++
Tendon pathology	−	+++	++	+++
Bone marrow edema	−	−	+	+++
Tophus or synovial vascularity	−	+++	−	+++
